# Fibromyalgia in systemic autoimmune rheumatic diseases: a mixed-methods study of patient and clinician perspectives

**DOI:** 10.1007/s00296-026-06143-y

**Published:** 2026-06-04

**Authors:** Dorsa Manavi, Alice Tunks, Wendy Diment, Alessandra Bortoluzzi, James A. Bourgeois, Lucy Calderwood, Xiaofeng Yan, Melanie Sloan

**Affiliations:** 1 University of Cambridge School of Clinical Medicine, Hills Rd, Cambridge, CB2 0SP UK; 2 Department of Public Health and Primary Care Unit, University of Cambridge, Cambridge, UK; 3 Patient and Public Co-Investigators, Cambridge, UK; 4 Department of Medical Sciences, Rheumatology Unit, University of Ferrara and Azienda Ospedaliero-Universitaria S. Anna, Ferrara, Italy; 5 Department of Psychiatry and Behavioral Sciences, Davis Medical Center, University of California, Sacramento, CA USA; 6 NorCal Neurostimulation, Folsom, CA USA; 7 Department of Dermatology, School of Medicine, University of California, Davis, CA USA; 8 Faculty of Medicine and Health Sciences, University of East Anglia, Norwich, England

**Keywords:** Fibromyalgia, Autoimmune diseases, Rheumatic diseases, Physician–patient, Relations, Diagnostic uncertainty, Mixed methods research, Surveys and questionnaires

## Abstract

Fibromyalgia is frequently diagnosed alongside systemic autoimmune rheumatic diseases (SARDs), yet impacts remain poorly understood. This study aimed to examine patients’ and clinicians’ perspectives of the psychosocial, clinical, and relational impact of fibromyalgia as a co-diagnosis in SARDs. We employed explanatory mixed-methods study integrating survey data from 1269 adults with SARDs, and interviews with patients (n = 19) and clinicians (n = 21). Outcomes included wellbeing (WEMWBS, 14–70), trust, confidence in receiving medical help, adaptation, satisfaction with care (all 1–5), and self-rated health (0–100). Independent t-tests compared outcomes by fibromyalgia status and by patients’ diagnostic acceptance. Qualitative open-text responses and interviews were analysed thematically. Among participants (95.1% female n = 1207; lupus 31.0% n = 393 and rheumatoid arthritis 31.3% n = 397), fibromyalgia was reported by 25.9% (n = 329) of whom 52.8% (n = 174) questioned diagnostic accuracy. Compared with those without fibromyalgia, affected participants reported significantly lower wellbeing (mean difference(SD) = -4.206(0.608)), trust in GPs (mean difference(SD) = -0.239(0.092)) and rheumatologists (mean difference(SD) = -0.515(0.097)), confidence in receiving help (mean difference(SD) = -0.459(0.073)), and satisfaction with care (mean difference(SD) = -0.563(0.073)), alongside poorer adaptation (mean difference(SD) = -0.223(0.066)) and life satisfaction (mean difference(SD) = -0.519(0.071)), but higher self-rated health (mean difference(SD) = 11.107(1.411); all p < 0.05). Diagnostic acceptance showed minimal quantitative differences across outcomes. Thematic analysis highlighted that diagnostic uncertainty was driven by overlapping symptoms and limited biomarkers. Variations in clinician practices affected patients’ trust and acceptance. Furthermore, misdiagnosis and dismissal led to stigma, delayed recognition of conditions, and considerable patient distress. In SARDs, fibromyalgia diagnosis is associated with poorer wellbeing and reduced trust in healthcare providers, irrespective of diagnostic acceptance, and often reflects diagnostic uncertainty. Mechanism-based explanations may help improve communication and provide greater clinical utility than categorical diagnostic labelling.

## Introduction

Fibromyalgia is a chronic pain condition characterised by widespread musculoskeletal pain, fatigue, cognitive dysfunction, and sleep disturbance with recorded prevalences of 2–3% [1]. Prevalence is markedly higher in those with systemic autoimmune rheumatic diseases (SARD) at rates of 10–30% [2]. Its pathophysiology remains unclear, with no definitive biomarkers or objective diagnostic tests [3]. A combination of genetic, hormonal, immunological, and environmental influences has been proposed to contribute its development [3]. Yet, without clear markers or a disease model, and the removal of tender-point examination from diagnostic criteria [4], the diagnosis is currently one of exclusion [5].

Overlapping symptom presentation, particularly of pain, fatigue, and cognitive dysfunction, make it challenging to distinguish fibromyalgia from SARDs or treatment-related side-effects [6]. Misclassification of fibromyalgia is estimated to be around 15% [7]. Relatedly, the diagnosis is suggested to alter how clinicians view patients [8], and influence how society perceives patients, impacting trust, communication, and self-understanding [9, 10].

The controversy of fibromyalgia can be traced to its history. Early in the twentieth century, similar symptom constellations were described as fibrositis, implying inflammatory pathology [11]. By mid-century, psychogenic rheumatism gained prominence, applied primarily to women presenting with diffuse pain, fatigue, and stiffness in the absence of objective pathology [12]. This attribution of physical symptoms to psychological distress positioned chronic pain within psychiatric discourse. Although subsequent research has identified physiological alterations in pain processing [13, 14], treatments remain heavily weighted toward psychological interventions [15], with modest efficacy [16].

Formal diagnostic criteria for fibromyalgia were introduced by the American College of Rheumatology in 1990, focusing on chronic widespread pain and the presence of ≥ 11 of 18 specified tender-points. The tender-point examination was later replaced in 2010/2011 by the Widespread Pain Index and Symptom Severity Scale, incorporating fatigue, cognitive dysfunction, and sleep disturbance. The 2016 update refined thresholds and clarified that fibromyalgia may be diagnosed alongside other conditions when symptoms cannot be better explained by another disorder [4].

Whilst fibromyalgia is frequently co-diagnosed in patients with SARDs [17], the psychosocial and clinical consequences of this co-diagnosis are poorly understood. This mixed-methods study investigates the acceptance and impact of fibromyalgia as a co-diagnosis among individuals with SARDs.

## Methods

### Design and measures

Data were collected in the LISTEN survey; an online mixed-methods survey distributed in 2020 to individuals with rheumatological diseases. The survey was developed as part of a wider programme of mixed-methods research in SARDs. The survey explored various aspects of patients' lives and medical care, including their experiences with fibromyalgia. Participants were recruited through a survey link distributed by patient organisations. Eligibility required age ≥ 18 years and self-reported clinical rheumatological disease diagnosis. Diagnoses, including fibromyalgia status, were self-reported and were not independently validated against clinical records or classification criteria. Full details of the survey development, design and recruitment are described in an earlier paper [18].

The survey included several self-reported measures. Mental wellbeing was assessed using the 14-item Warwick Edinburgh Mental Wellbeing Scale (WEMWBS) [19], yielding scored from 14 to 70. Trust in General Practitioners (GP) and rheumatologists, and confidence in receiving required medical help, were rated on a 5-point Likert scale. Adaption to chronic disease was assessed using the ADAPT instrument [20], a patient-designed 5-point Likert scale, measuring life satisfaction, satisfaction with medical care, disease adaptation, perceived control and knowledge. Self-reported disease activity was measured on a 0–100 scale, with higher scores indicating greater severity.

Qualitative data were collected by interviews with clinicians and patients and using an open-text question "Please write any more information about why you feel the fibromyalgia diagnosis is correct/incorrect". Interviewees were purposively sampled to ensure a range of sociodemographic characteristics.

Ethical approval was obtained from the Cambridge Psychology Research Ethics Committee (PRE.2019.099 on 30 Jan 2020). All participants provided informed consent electronically through the online surveys on Qualtrics and/or verbally before interviews.

### Quantitative data analysis

Survey data were analysed using IBM SPSS Statistics. Descriptive statistics presented the percentages of participants who viewed their diagnosis as accurate or misdiagnosed. Independent samples t-tests compared parameters across participants with and without a fibromyalgia diagnosis, and between those who accepted versus rejected their diagnosis; participants unsure of diagnostic accuracy were excluded from the latter analyses. For each measure, mean scores were compared, with statistical significance set at p = 0.05. All analyses were conducted after approval of the Statistical Analysis Plan to minimise bias.

### Qualitative data analysis

Data were analysed using thematic analysis [21]. An inductive approach was used, with repeated reading to achieve familiarisation with the data, followed with iterative line-by-line coding and ongoing annotation. Coding involved constant comparison across the dataset and continued until no new codes were identified. Initial codes were organised into preliminary themes, which were subsequently reviewed, refined, and grouped into overarching themes through ongoing discussion within the research team. The dataset was re-examined to ensure consistent and comprehensive representation of the data within the final thematic structure.

## Results

### Quantitative results

#### Descriptive Statistics and Diagnostic Patterns

Of the 1269 participants reporting fibromyalgia status (Table 1), 25.9% (n = 329) reported a fibromyalgia diagnosis, of whom 97.0% self-identified as women and 2.7% as men. The remaining 74.1% (n = 940) reported no fibromyalgia diagnosis. Among those diagnosed, 47.1% (n = 155) accepted the diagnosis, 19.1% (n = 63) believed another condition better explained their symptoms, 9.1% (n = 30) believed they were misdiagnosed for other reasons, and 24.6% (n = 81) were unsure. Overall, 52.8% expressed either doubt or uncertainty regarding the diagnosis.

**Table 1:** Participant characteristics

Characteristic	Patient survey (n = 1269) (%)	Patient interviews	Clinician interviews (n = 21) (%)
		(n = 19) (%)	
*Age*			
18- 30	68 (5.4%)	2 (11%)	0
30–39	144 (11.3%)	2 (11%)	7 (33%)
40–49	229 (18.0%)	5 (26%)	7 (33%)
50–59	384 (30.3%)	3 (16%)	4 (19%)
60–69 (60 + for clinicians)	294 (23.2%)	3 (16%)	3 (14%)
70 +	150 (11.8%)	2 (11%)	N/A
Prefer not to say	0	0	0
*Gender*			
Female	1207 (95.1%)	15 (79%)	12 (57%)
Male	56 (4.4%)	2 (11%)	9 (43%)
Non-binary	3 (0.2%)	0	0
Prefer not to say	3 (0.2%)	0	0
*Disease*			
SLE	393 (31.0%)	4 (21%)	NA
Rheumatoid/Inflammatory arthritis	397 (31.3%)	4 (21%)	NA
Vasculitis	46 (3.6%)	1 (5%)	NA
Sjögrens	114 (9.0%)	2 (11%)	NA
UCTD	43 (3.4%)	2 (11%)	NA
Myositis	44 (3.5%)	1 (5%)	NA
Systemic sclerosis	97 (7.6%)	1 (5%)	NA
Mixed/multiple/Overlap	88 (6.9%)	2 (11%)	NA
Other	47 (3.7%)	0	NA
*Clinician Role*			
Rheumatologist	NA	NA	8 (38%)
Psychiatrist	NA	NA	3 (14%)
Neurologist	NA	NA	3 (14%)
Rheumatology nurse	NA	NA	3 (14%)
GP/Primary care	NA	NA	4 (19%)
Other speciality	NA	NA	0

#### Time, healthcare, adaptation, and wellbeing in individuals with and without fibromyalgia

**Fig. 1:**
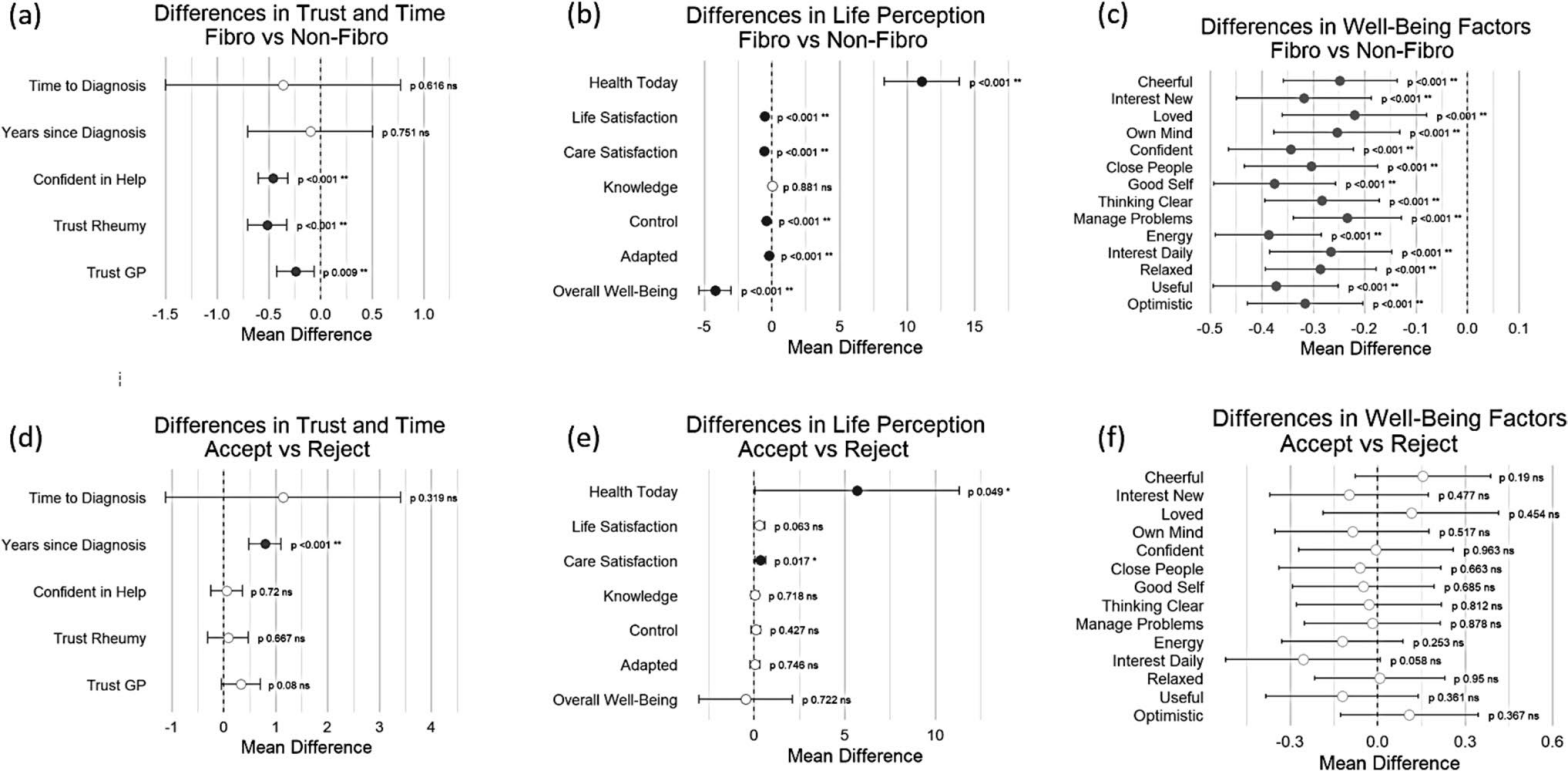
Forest plots showing group differences across psychosocial, health, and wellbeing domains. **a–c** Diagnosis vs. No Diagnosis comparisons: **a** time, trust, confidence in receiving help; **b** life perception and health factors; **c** wellbeing factors. **d–f** Accept vs. Reject comparisons: **d** time, trust, life perception, and wellbeing factors; **e** life perception and health factors; **f** wellbeing factors. Error bars represent 95% confidence intervals. Error bars represent 95% confidence intervals. All comparisons are based on independent t-tests. Shaded markers indicate statistically significant differences (p < 0.05), and exact p-values are displayed beside each estimate

Comparisons between participants with and without a fibromyalgia diagnosis were made across measures of time, trust, life perception and wellbeing (Table 2; Fig. 1a–c). No significant differences were observed in years to diagnosis or years since diagnosis of the primary autoimmune condition. Participants with fibromyalgia reported significantly lower levels of confidence in receiving help (mean difference(SD) = -0.459(0.073), p < 0.001), and significantly less trust in both GPs (mean difference(SD) = -0.239(0.092), p = 0.009) and rheumatologists (mean difference(SD) = -0.515(0.097), p < 0.001) (Table 2a; Fig. 1a).

Participants with fibromyalgia reported significantly better current health (mean difference(SD) = 11.107(1.411), p < 0.001) than those without the diagnosis (Table 2a; Fig. 1b). They reported significantly lower levels of satisfaction with both life (mean difference(SD) = -0.519(0.071), p < 0.001) and the medical care they received (mean difference(SD) = -0.563(0.073), p < 0.001), life adaptation (mean difference(SD) = -0.223(0.066), p < 0.001) and perceived control (mean difference(SD) = -0.397(0.064), p < 0.001) (Table 2a; Fig. 1b). No significant differences were found in self-reported knowledge between groups.

Participants with fibromyalgia also reported significantly poorer scores across all wellbeing items (Table 2b; Fig. 1c), culminating in a significantly lower total well-being score (mean difference(SD) = -4.20634(0.60843), p < 0.001).

**Table 2:** Comparison of time, healthcare, adaptation, health, and wellbeing by fibromyalgia status

a) Time variables, healthcare experience, adaptation items, and current disease activity (health today). N ranged from 958 (min) to 1269 (max)							
Variable/Items	t	Mean Difference	std. error difference	CI Lower	CI Upper	p value	Cohen’s d
*Time Variables (years)*							
Time to Diagnosis	-0.622	-0.361	0.579	-1.498	0.776	0.616	0.032
Years since Diagnosis	-0.317	-0.097	0.307	-0.701	0.506	0.751	0.020
*Healthcare Experience (1–5)*							
Confident in Help	-6.298	-0.459	0.073	-0.602	-0.316	** < 0.001**	0.418
Trust GP	-2.603	-0.239	0.092	-0.42	-0.059	**0.009**	0.174
Trust Rheumy	-5.318	-0.515	0.097	-0.705	-0.325	** < 0.001**	0.380
*Adaptation Items (1–5)*							
Adapted	-3.357	-0.223	0.066	-0.353	-0.093	** < 0.001**	0.220
Control	-6.190	-0.397	0.064	-0.522	-0.271	** < 0.001**	0.375
Knowledge	0.150	0.009	0.057	-0.103	0.120	0.881	0.010
Life Satisfaction	-7.307	-0.519	0.071	-0.659	-0.380	** < 0.001**	0.468
Care Satisfaction	-7.668	-0.563	0.073	-0.707	-0.419	** < 0.001**	0.491
Health Today (1–100)	7.870	11.107	1.411	8.336	13.878	** < 0.001**	0.463

b) Warwick–Edinburgh Mental Wellbeing Scale (WEMWBS) overall score and individual items. N ranged from 1261 (min) to 1269 (max)							
Variable	t	Mean Difference	std. error difference	CI Lower	CI Upper	p value	Cohen’s d
Overall Well-Being (14–70)	-6.913	-4.20634	0.60843	-5.39998	-3.01269	** < 0.001**	0.444
*Individual Items (1–5)*							
Optimistic	-5.514	-0.316	0.057	-0.428	-0.203	** < 0.001**	0.356
Useful	-6.023	-0.372	0.062	-0.494	-0.251	** < 0.001**	0.391
Relaxed	-5.224	-0.286	0.055	-0.393	-0.178	** < 0.001**	0.336
Interest Daily	-4.385	-0.266	0.061	-0.385	-0.147	** < 0.001**	0.282
Energy	-7.347	-0.387	0.053	-0.490	-0.284	** < 0.001**	0.439
Manage Problems	-4.378	-0.234	0.053	-0.339	-0.129	** < 0.001**	0.281
Thinking Clear	-5.018	-0.283	0.056	-0.394	-0.172	** < 0.001**	0.322
Good Self	-6.232	-0.375	0.060	-0.493	-0.257	** < 0.001**	0.400
Close People	-4.630	-0.304	0.066	-0.434	-0.175	** < 0.001**	0.308
Confident	-5.551	-0.344	0.062	-0.465	-0.222	** < 0.001**	0.360
Own Mind	-4.082	-0.254	0.062	-0.376	-0.132	** < 0.001**	0.274
Loved	-3.077	-0.220	0.071	-0.360	-0.079	** < 0.001**	0.205
Interest New	-4.755	-0.318	0.067	-0.449	-0.187	** < 0.001**	0.306
Cheerful	-4.405	-0.248	0.056	-0.358	-0.137	** < 0.001**	0.283

Bold values indicate statistically significant results (p < 0.05)

#### The impact of accepting versus rejecting fibromyalgia diagnosis.

Among participants with fibromyalgia, comparisons were made between those who accepted their diagnosis (n = 155) and those who felt misdiagnosed (n = 93) (Table 3; Fig. 1d–f). Patients who accepted their diagnosis had lived with SARDs diagnosis longer (mean difference(SD) = 0.794(0.157), p < 0.001) (Table 3a; Fig. 1d) and reported better current health (mean difference(SD) = 5.684(2.861), p = 0.049) (Table 3a, Fig. 1e). They also felt more satisfied with their care (mean difference(SD) = 0.359(0.150), p = 0.017) (Table 3a; Fig. 1e). No other significant differences were observed between groups, including in ratings of medical care (Table 3a; Fig. 1d) or wellbeing (Table 3b; Fig. 1f).

**Table 3:** Comparison of time, healthcare, adaptation, health, and wellbeing by fibromyalgia diagnosis acceptance

a) Time variables, healthcare experience, adaptation items, and current disease activity (health today). N ranged from 201 (min) to 248 (max)							
Variable/Items	t	Mean Difference	std. error difference	CI Lower	CI Upper	p value	Cohen’s d
*Time Variables (years)*							
Time to Diagnosis	0.998	1.144	1.147	-1.115	3.403	0.319	0.131
Years since Diagnosis	5.042	0.794	0.157	0.483	1.104	** < 0.001**	0.703
*Healthcare Experience (1–5)*							
Confident in Help	0.359	0.056	0.156	-0.251	0.362	0.720	0.047
Trust GP	0.364	0.332	0.189	-0.04	0.704	0.080	0.239
Trust Rheumy	0.205	0.085	0.197	-0.304	0.474	0.667	0.062
*Adaptation Items (1–5)*							
Adapted	0.324	0.045	0.139	-0.229	0.319	0.746	0.043
Control	0.796	0.103	0.13	-0.152	0.359	0.427	0.104
Knowledge	0.362	0.039	0.107	-0.172	0.250	0.718	0.047
Life Satisfaction	1.869	0.282	0.151	-0.015	0.579	0.063	0.245
Care Satisfaction	2.398	0.359	0.150	0.064	0.654	**0.017**	0.314
Health Today (1*–*100)	1.987	5.689	2.861	0.035	11.333	**0.049**	0.274

b) Warwick–Edinburgh Mental Wellbeing Scale (WEMWBS) overall score and individual item. N ranged from 246 (min) to 248 (max)							
Variable	t	Mean Difference	std. error difference	CI Lower	CI Upper	p value	Cohen’s d
Overall Well-Being (14–70)	-0.356	-0.4649	1.30497	-3.03535	2.10556	0.722	0.047
*Individual Items (1–5)*							
Optimistic	0.904	0.109	0.120	-0.128	0.346	0.367	0.119
Useful	-0.915	-0.122	0.133	-0.384	0.140	0.361	0.120
Relaxed	0.063	0.007	0.113	-0.216	0.230	0.950	0.008
Interest Daily	-1.903	-0.256	0.135	-0.521	0.009	0.058	0.250
Energy	-1.146	-0.121	0.106	-0.330	0.087	0.253	0.151
Manage Problems	-0.153	-0.018	0.118	-0.251	0.215	0.878	0.020
Thinking Clear	-0.238	-0.030	0.126	-0.278	0.218	0.812	0.031
Good Self	-0.407	-0.050	0.123	-0.293	0.193	0.685	0.053
Close People	-0.436	-0.061	0.141	-0.339	0.216	0.663	0.057
Confident	-0.047	-0.006	0.135	-0.271	0.259	0.963	0.006
Own Mind	-0.648	-0.087	0.134	-0.352	0.177	0.517	0.085
Loved	0.749	0.115	0.153	-0.187	0.416	0.454	0.099
Interest New	-0.712	-0.098	0.138	-0.370	0.174	0.477	0.094
Cheerful	1.314	0.155	0.118	-0.077	0.387	0.190	0.173

Bold values indicate statistically significant results (p < 0.05)

### Qualitative results

#### Theme 1: diagnostic uncertainty and challenges in differentiation

Participants (ppt) consistently expressed difficulty distinguishing fibromyalgia from their SARD(s). Diagnostic ambiguity emerged from overlapping symptoms, absence of definitive biomarkers, and non-specific diagnostic criteria.

##### Subtheme 1.1: overlapping clinical symptoms

Many patients reported symptoms that could be attributed to fibromyalgia, autoimmune condition, or treatment side-effects. Overlapping features such as pain, fatigue, and cognitive difficulties created uncertainty, leading some to question the fibromyalgia diagnosis as redundant: “*Sore tendons, muscle pain and chronic fatigue are all signs of a lupus flare for me and other lupus patients*.” ppt627, lupus, 50 s.

Others felt able to distinguish fibromyalgia symptom from those of SARDs: “*I already recognised the symptoms as they were different from my lupus ones*.” ppt768, lupus, 40 s.

##### Subtheme 1.2: lack of clear diagnostic tests

Participants frequently commented on the absence of reliable diagnostic markers for fibromyalgia: “*The symptoms of fibro mirror those of Lupus and I question how a diagnosis can be made when no tests have been done*.” ppt1679, Sjögren’s, 50 s.

Many patients reported that diagnostic decisions on fibromyalgia appeared to hinge disproportionately on blood test results, despite symptoms suggesting alternative diagnoses: “*Blood tests are the only thing they bother with. Swollen joints and other physical, visible symptoms ignored because of negative blood tests*.” ppt472, scleroderma, 50 s.

This reliance on exclusion, particularly when autoimmune markers were negative or inconclusive, often led participants to perceive fibromyalgia as a fallback label: “*My lupus is seronegative and I believe fibro was an 'easy' diagnosis*.” ppt1463, lupus, 30 s.

The perceived vagueness of diagnostic criteria was echoed by clinicians, with one reflecting on the difficulty of applying fibromyalgia criteria: “*There are some guidelines but they change a lot(…) it is difficult to keep up*.” ppt21, GP.

Although official guidelines have moved away from tender-point testing, those assessed using tender-points often found the diagnosis more acceptable: “*I have pain in most of the places they check, so I believe I meet the diagnostic criteria for fibromyalgia*.” ppt365, UCTD, 50 s.

##### Subtheme 1.3: diagnostic timing and autoimmune disease control

Some participants reflected on the timing of their fibromyalgia diagnosis in relation to autoimmune disease activity. The diagnosis was more acceptable when their autoimmune condition was stable or well controlled but was questioned when made during periods of active or poorly controlled disease: “*My RA was so out of control that it would be difficult to say if correct or not.*” ppt33, rheumatoid arthritis, 50 s. 

#### Theme 2: Variability in Healthcare Providers’ Knowledge and Practices

Participants reported variability in clinicians’ understanding, attitudes, and diagnostic practices regarding fibromyalgia, which was felt to undermine trust, diagnostic clarity, and engagement with care.

##### Subtheme 2.1: conflicting medical interpretations of fibromyalgia’s nature

Clinicians varied in their conceptualisation of fibromyalgia, describing neurological, post-viral, stress-related, or depressive origins, while others questioned its existence as a distinct entity, viewing it instead as part of inflammatory disease spectra. Patients described diagnostic instability, with diagnoses shifting according to individual clinicians’ beliefs (see Table 4a).

**Table 4:** Representative Quotes for Theme 2: Variability in Healthcare Providers’ Knowledge and Practices

a) Healthcare Providers’ Knowledge and Practices and Patient’s Reported Experiences	
Clinicians’ Perspectives	Patients’ Reported Experiences
“Pain processing might be a bit different to what is healthy and normal” ppt11, rheumatology registrar (specialist trainee) “Some of it is their coping mechanism” ppt 17, rheumatologist “Where does fibromyalgia come from? It’s the brain.” ppt 12, neurologist “It’s re-education of your muscles and pain pathways” ppt 6, nurse “We are not convinced that fibromyalgia actually exists (…) you need to think of these things as being on a spectrum rather than separate conditions.” ppt 27, rheumatologist	“Diagnoses 'shifted’ and changed depending on the views of different doctors” ppt1209, lupus, 60 s “My symptoms sometimes appear to be fibromyalgia but if it is fibro it isn’t constant and differs depending on which medical professionals sees you” ppt517, other, 50 s “I was told at my first appointment with my rheumatologist that I had elements of fibromyalgia. This has not been mentioned since.” ppt15, rheumatoid arthritis, 50 s “Was not my regular doctors who suggested it. My blood results for inflammation are lower than normal. My regular doctors are aware of this.” ppt203, rheumatoid arthritis, 70 + “It was suggested by the rheumatology nurse but dismissed by the consultant” ppt348, rheumatoid arthritis, 40 s

b) Role of Communication and Diagnostic Framing in Patient Acceptance	
Clinicians’ Reported Experiences	Patients’ Reported Experiences
“I say I think it’s fibromyalgia because you’ve got these features, not because I don’t know what’s wrong with you and that usually works.” ppt 4, rheumatologist “some people have had very bad experiences, they list all the people they’ve seen and have taken away the perception that they said it was all in my head (…) we may have said the same diagnosis but with a better use of language and more empathy and connection to that individuals life, they are often fine about it.” ppt 11, rheumatology registrar (specialist trainee)	“My consultant diagnosed it. At first I doubted it, but he spent quite a long time explaining it to me. Also convincing me these symptoms weren't in my head. But I still don't mention it to my doctor.” ppt887, other, 60 s “He downgraded me to "lupus like syndrome" and fibromyalgia (…) This was extremely distressing as I found out via his post review letter to my GP.” ppt293, other, 50 s

*Ppt*  participant; quotes include participant number, role/condition, and age group where applicable

Clinicians also varied widely in their capacity to understand and engage with the lived experience of chronic pain. One clinician demonstrated deep empathy for what it means to live with unexplained symptoms, stressing the emotional impact of being dismissed: *“They’re going to go home and they’re going to live with these symptoms not knowing why(…) We need to think as if we were the patient(…) Sometimes that is to listen, to talk, to reassure*” ppt27, rheumatologist.

In contrast, another clinician framed the absence of objective disease as positive, with less acknowledgement of the impact of ongoing pain: “*Tell them it’s good news(…) they are going to have to learn to live with the pain*.” ppt4, rheumatologist.

##### Subtheme 2.2: psychological framing and stigma undermine patient trust

Patients reported distress when symptoms were attributed to fibromyalgia, particularly when serious physical pathology was later identified: “*Demonstrated loss of coordination was thought to be put on(…) some kind of cry for help due to trauma(…) I was re-diagnosed with fibromyalgia after this. The loss of memory, headaches, loss of coordination have since been proven to be due to SLE lupus damage to the brain*” ppt627, lupus, 50 s.

Clinicians acknowledged difficulty discussing mental health without implying a psychogenic origin: “*the more time I spend talking about [mental health] the more they are thinking I’m just classing them as a loony and that it’s just all in their head. We have a real issue with people with somatisation, medically unexplained symptoms, hypochondriasis and health anxiety(…) fibromyalgia there’s a mental health component(…)*” ppt5, GP.

Several clinicians perceived that fibromyalgia was stereotypically linked to a certain mindset, in contrast to other conditions: “*the thing with fibromyalgia, people think, doctors think, they are not the kind of people who would get fibromyalgia. So people think I might possibly get lupus or rheumatoid arthritis but I’m not the kind of the person with the mentality to get fibromyalgia*” ppt4, rheumatologist.

Participants reported greater acceptance of the diagnosis when explanations were clear and collaborative, while abrupt diagnostic shifts or poor communication undermined trust. Clinicians similarly noted that how the diagnosis is framed influences patient acceptance (see Table 4b).

#### Theme 3: impact of diagnosis, misdiagnosis, and medical dismissal

Patients described mixed experiences following a fibromyalgia diagnosis. Many perceived fibromyalgia as a diagnosis applied in the absence of clear alternatives, contributing to mistrust, distress, and feelings of dismissal. Misdiagnosis and diagnostic inertia were reported to delay identification of other conditions, sometimes by years (see Table 5 for all additional theme 3 quotations).

**Table 5:** Additional Participant Quotations Illustrating Theme 3: Impact of Diagnosis, Misdiagnosis, and Medical Dismissal

Subtheme		Representative Quotes
3.1	Acceptability of Diagnosis May Result in Feelings of Relief and Validation	"All of the additional symptoms like sensitivity to light and loud noises make complete sense." ppt270, Rheumatoid Arthritis, 30 s "I get unexplained pain all over my body which couldn’t be explained by lupus alone. It was like burning areas randomly on my body." ppt833, other, (18–29),
3.2	Fibromyalgia is Perceived as a ‘Catch-All’ or ‘Dustbin’ Diagnosis, with Significant Emotional Toll of Misdiagnosis and Dismissal	“It was never followed up on just suggested by a GP I hadn’t seen before” ppt169, lupus, 50 s, “It was a lazy diagnosis made by a junior Dr who didn't even have my full symptoms to hand. It was rebuked a day later by my consultant but not before causing me distress.” ppt1330, lupus, 40 s "It’s a bucket diagnosis given when blood tests don’t fit the symptoms(…)" ppt197, vasculitis, 50 s "Seems to be an umbrella for nonspecific symptoms." ppt684, other, 50 s "I received a fibromyalgia diagnosis around a year after my lupus one. I felt it was used as a cop-out(…) I have never been able to distinguish between lupus symptoms or fibromyalgia symptoms." ppt825, other, 30 s "I felt the GP was unsure when x-rays ruled out osteoporosis and chose fibromyalgia as a catch-all diagnosis." ppt1389, other, 60 s "Issues with brain fog were put down to fibromyalgia, but I’m aware that they are also a symptom of Lupus." ppt152, MCTD, 30 s
3.3	Diagnosis of Fibromyalgia Can Lead to Dismissal of Future Symptoms	“My current Rheumatologist puts too much down to Fibro, rather than concentrating on my SLE & almost missed Toxic Retinitis diagnosis…” ppt 1147, lupus, 50 s "I feel the fibromyalgia diagnosis was an add-on so that they wouldn’t have to treat my symptoms." ppt709, MCTD, 50 s "Think it was given to keep me quiet rather than investigate further." ppt210, rheumatoid arthritis, 30 s "It was a deliberate attempt by my rheumatologist to kick me off the service and not deal with my Sjogren's and lupus symptoms (…)" ppt 975, other, 30 s “If someone has a label of fibromyalgia, before they are given that diagnosis they’ve usually had the full range of tests, x-rays from the rheumatologist and they’ve not found anything then if, erm but they get that fibromyalgia diagnosis then things might change in the future, but it’s very hard to step back from that labelling and go let’s reassess and look again. Also I think it’s very stressful for patients to have that diagnosis because they don’t know what to do, but it’s hard from a clinician's perspective because you don’t want to keep requesting tests, and x-rays when that’s not necessarily what’s going to help them.” ppt 6, nurse “I have experienced this diagnosis was wrong on at least 3 accounts… the bombardment of fibromyalgia/mental health support over several years was more than insulting. The medical professionals' inability to listen to my concerns thoroughly has resulted in me developing additional health complications.” ppt 1061, scleroderma, 50 s, “I was, essentially, issued with 'rubbish' diagnoses: thrown in a diagnostic dustbin: ignored and dismissed.” ppt 1209, lupus, 60 s “I feel completely cut adrift and abandoned by my rheumatologist who usually spoke with me every 3 months (…) mind you, he stopped listening to the issues I had, and became dismissive.” ppt 293, other, 50 s
3.4	Patients reported needing to rely on self-advocacy and private healthcare to receive help	"I’ve sometimes ended up having to seek second opinions and private treatment for my ongoing symptoms." ppt 884, rheumatoid arthritis, 30 s "A private Rheumatologist found what was going on (had private health cover through my work back then)." ppt 888, rheumatoid arthritis, 50 s

*Ppt* = *participant; quotes include participant number, role/condition, and age group where applicable*

##### Subtheme 3.1: acceptability of diagnosis may result in feelings of relief and validation

For some, fibromyalgia offered an explanation for otherwise unexplained symptoms, providing reassurance and validation: "*I’ve had a lot of pain in my body not usually connected to RA so it was a relief to know it was fibromyalgia*." ppt1629, rheumatoid arthritis, 60 s.

##### Subtheme 3.2: fibromyalgia as a ‘Catch-All’ diagnosis with emotional cost

Fibromyalgia was felt to be prematurely diagnosed at times, especially when patients interacted with a new physician or ones that were unaware of their previous diagnosis: “*The diagnosis was made before the physician had all the information(…) after a telephone assessment by someone who had never met me, had not seen my scan results, after I'd had scans that led to my eventual diagnosis*.” ppt310, rheumatoid arthritis, 40 s.

Many described fibromyalgia as a convenient label used to avoid further investigation, justify discharge from specialist care, or due to diagnostic fatigue: "*Fibro is a made up word for a collection of symptoms that don't match with blood results so doctors give the ‘fibro’ diagnosis to shut you up and go away*" ppt91, lupus, 40 s.

Greater patient knowledge of autoimmune disease led some to question the accuracy of fibromyalgia diagnosis: "*All my pain is [in] joints rather than muscle. I have strongly positive ANA, CRP and ESR and visibly swollen joints*." ppt383, other, 50 s.

##### Subtheme 3.3: diagnosis of fibromyalgia can lead to dismissal of future symptoms

Participants reported that once fibromyalgia was diagnosed, clinicians were less likely to pursue further investigations, with new or worsening symptoms attributed to fibromyalgia without assessment: “*Signs and symptoms that pointed towards other potential underlying medical conditions (e.g. sporadic appearance of 'text book' malar rash) were routinely dismissed and written off with 'oh, that’s the fibromyalgia…no treatment for that…nothing I can do'. And this without any clinical examination or further investigations*.” ppt1209, lupus, 60 s.

In some cases, this led to serious harm and delayed diagnosis: “*I barely survived a renal flare due to assumptions about fibromyalgia and what doctors assumed was going on with me psychologically without ever respectfully consulting me on the issue once*.” ppt627, lupus, 50 s.

##### Subtheme 3.4: patients reported needing to rely on self-advocacy and private healthcare to receive help

Participants described needing to self-advocate or challenge diagnoses: "*If I gave up, I'd be stuck with fibromyalgia*." ppt91, lupus, 40 s.

Some pursued private healthcare to obtain revised diagnoses: "*I was misdiagnosed by my GP. I had to pay to see a private GP, then a rheumatologist. I was diagnosed with polymyalgia/giant cell arteritis*." ppt166, lupus, 70 + 

## Discussion

This study explored the implications of a fibromyalgia co-diagnosis among individuals with SARDs. Approximately one quarter (25.9%) reported a fibromyalgia diagnosis, and over half of these (52.8%) questioned its accuracy. Those with a co-diagnosis reported poorer wellbeing, lower trust in healthcare, and reduced satisfaction with life and care, alongside diminished perceived control and adaptation. This was despite rating their current health as better than participants without fibromyalgia.

Qualitative findings contextualised these patterns. Fibromyalgia was frequently interpreted as a poorly explained, default label applied when clinicians were uncertain, signalling diagnostic fatigue. Following the diagnosis, many patients reported increased difficulty accessing investigation, treatment, or follow-up, with some discharged from rheumatology care. Clinicians expressed divergent conceptualisations of fibromyalgia’s aetiology and varied in how they framed the diagnosis, ranging from central nervous system hypersensitivity to stress-related or coping-based mechanisms. Some individuals reported relief or validation on receiving a fibromyalgia co-diagnosis. However, for the majority, a fibromyalgia co-diagnosis reflected persistent, under-managed symptom burdens compounded by inconsistent communication and eroded trust.

These findings align with prior qualitative research demonstrating that initial relief at receiving a fibromyalgia diagnosis often gives way to disappointment when it offers limited understanding or therapeutic value, and in some cases contributes to identity disruption or social withdrawal [9, 22, 23]. Our results reinforce the concerns that fibromyalgia may function poorly as a coherent explanatory construct. Despite decades of research, no consistent biomarkers, pathognomonic features, or unified pathophysiological mechanisms have been established [3]. Increasingly, fibromyalgia is conceptualised as a heterogeneous syndrome encompassing multiple biological and psychosocial endotypes. Cluster and neuroimaging studies identify distinct subgroups with differing pain, cognitive-affective, and neurobiological profiles [24–26], likely underpinned by varied mechanisms [3, 27]. This heterogeneity limits reproducibility, contributes to the absence of universally effective treatments, and perpetuates clinical uncertainty and scepticism.

The markedly differing conceptualisation of fibromyalgia among clinicians in our study illustrates a lack of shared theoretical grounding. This mirrors wider literature in which some practitioners frame fibromyalgia as primarily biological, others as primarily psychosocial [28], and where physicians report uncertainty in diagnosis and management [29]. Mixed-model conceptualisations are increasingly recognised, combining biological vulnerability with psychosocial modulation [27, 30].

Our qualitative results suggests that these divergent frameworks lead to substantial variation in clinical approach and communication. Patients described diagnoses that shifted between clinicians and explanations that emphasised stress or coping. Even when well-intentioned, patients often interpreted such framing as minimisation, describing feelings of dismissal and erosion of trust. These interactions exemplify the mutual scepticism produced by diagnostic ambiguity: clinicians often feel uncertain or powerless, while patients perceive disbelief. As a result, the fibromyalgia diagnosis appears to serve primarily as an administrative tool, allowing clinicians to categorise unexplained symptoms without necessarily improving understanding, communication, or management. This allows patients to receive a diagnosis that legitimises their symptoms “on paper” yet simultaneously signals to both clinician and patient that further investigation or intervention is unlikely to be pursued. Similar dynamics have been described in primary care, where both fibromyalgia patients and clinicians report frustration, uncertainty, and unmet expectations [31].

Most existing literature focuses on individuals with a primary fibromyalgia diagnosis [32]. Few studies have explored co-diagnosis in SARDs, where fibromyalgia overlays complex, biologically validated diseases. Recent studies in inflammatory arthritis report substantial rates of fibromyalgia based on screening tools [33–35]; however, such measures may in part capture overlapping symptom burden in the context of active or inadequately controlled disease, rather than a clearly distinct comorbid condition. Our findings suggest the consequences differ: rather than legitimising symptoms, a fibromyalgia co-diagnosis can de-legitimise previously validated disease, reducing the perceived need for further investigation. Self-reported diagnoses may have influenced reported fibromyalgia prevalence and perceptions of diagnostic accuracy, potentially overestimating prevalence without clinical validation. However, as clinical care often relies on patient-reported diagnostic history, these reports also reflect real-world diagnostic communication. Participants could express uncertainty regarding diagnostic accuracy, and the high level of uncertainty observed suggests limitations in diagnostic clarity and communication. Reported misdiagnosis may therefore reflect this uncertainty rather than confirmed clinical error.

Widespread pain, fatigue, and cognitive dysfunction also occur in active SARDs due to the direct effect of disease [36], residual activity, treatment side-effects, or secondary nociplastic processes. For example, drug-induced myopathies are frequently reported with glucocorticoids and antimalarials [37]. These overlaps complicate symptom attribution and requires clinicians to distinguish multiple contributors, an inherently uncertain task [35]. Consequently, the fibromyalgia label may function less as a distinct comorbid entity and more as a marker of diagnostic limits, a view also reflected in ongoing debate regarding the purpose and clinical utility of fibromyalgia diagnostic criteria [38]. A mechanism-based approach, explicitly acknowledging possible contributors, could offer greater clinical utility than a categorical diagnosis with conceptually unstable boundaries.

Our qualitative results also suggest that beyond its diagnostic ambiguity, the fibromyalgia label carries substantial psychosocial implications, echoed in wider literature [39]. Disbelief and psychologisation can prompt social withdrawal and self-doubt regarding the legitimacy of their own suffering [32, 36]: Many described feeling discredited or ashamed, suggesting an element of internalised stigma rather than explicit self-blame [9]. This may explain our quantitative paradox: participants with a fibromyalgia diagnosis reported better perceived current health but poorer wellbeing, life satisfaction, and sense of control. This may reflect a reframing of symptoms, whereby individuals perceive their overall health as stable or non-progressive, while broader aspects of wellbeing remain adversely affected. Our qualitative findings suggest that this may arise when persisting symptoms are reattributed to fibromyalgia rather than active disease, shifting the perceived locus of responsibility towards patients and shaping how health and wellbeing are evaluated.

The gendered history of fibromyalgia likely exacerbates these dynamics. Fibromyalgia remains predominantly diagnosed in women, a pattern that has historically invited psychologisation and trivialisation [40, 41]. A recent metasynthesis highlighted how women’s pain is frequently minimised or reframed as emotional distress [9]. This aligns with evidence of enduring gender disparities in pain assessment and management [42]. Clinicians’ emphasis on stress and coping in this study, though often intended to be supportive, may inadvertently reproduce these gendered assumptions and reinforce patients’ experiences of dismissal.

### Clinical implications and future research

Our findings underscore the urgent need for transparent communication and mechanism-based clinical reasoning rather than reliance on diagnostic labelling. Clinicians must explicitly acknowledge diagnostic uncertainty and actively consider the multiple mechanisms contributing to patients’ symptoms, including inflammatory activity, nociplastic processes, medication effects, and psychosocial factors. Collaborative, explanatory dialogue is essential to maintain therapeutic relationships and ensure ongoing engagement with care. Failure to do so risks eroding trust, disrupting continuity of care, and leaving symptoms under-treated. Educational initiatives should prioritise helping clinicians identify fibromyalgia-type symptom clusters as markers of unmet symptom burden and ongoing suffering, rather than defaulting to fibromyalgia labelling without careful, mechanism-informed consideration.

Future research should explore mechanism-based phenotyping in SARD patients with fibromyalgia-type symptoms and evaluate interventions to improve clinician-patient communication and reduce stigma.

Above all, clinical care must prioritise symptom management and quality of life. Regardless of diagnostic uncertainty or labelling, patients with persistent pain, fatigue, and cognitive symptoms require active management and ongoing support to achieve an acceptable quality of life.

### Strengths and limitations

This mixed-methods study integrates patient and clinician perspectives within an under-studied context and draws on a relatively large sample. Limitations include reliance on self-reported diagnoses, which were not independently validated and may introduce misclassification bias. Objective disease activity measures were not included, limiting clinical contextualisation of symptom burden. However, the study aimed to capture patient-reported experiences, which may not be fully reflected by standard disease activity indices, particularly in the presence of persistent symptoms despite controlled disease. The predominantly female sample limits generalisability. Participants were recruited in part through patient organisations and may therefore represent a more engaged, health-literate, or advocacy-oriented group, introducing potential selection bias. Analyses were descriptive and unadjusted for potential confounders; findings should therefore be interpreted as associations rather than causal relationships.

## Conclusion

While challenges surrounding fibromyalgia as a diagnosis are well recognised, this study demonstrates that these issues acquire particular significance in the context of SARDs. Here, the fibromyalgia label often marks diagnostic and therapeutic uncertainty, disrupts continuity of care, and undermines the therapeutic relationship, with lasting consequences for patients’ quality of life and future healthcare interactions. A mechanism-based, communicatively transparent approach may therefore offer greater explanatory and therapeutic value than categorical labelling, supporting more nuanced, empathic, and biopsychosocially informed care. 

## Data Availability

Anonymised quantitative data will be made available on reasonable request following completion of the analysis for all related studies. Qualitative data such as interview transcripts will not be made available due to its potentially identifying nature.

## Declarations

### Patient and public partnership

Patients were actively involved throughout every stage of this study, including contributing to the development of the research question and survey to ensure relevance to patient priorities and lived experience. Patients participated in the study through completion of surveys and qualitative interviews. Two patient partners are co-authors on this manuscript and contributed to study interpretation and reporting. The findings will be disseminated to patients and the public through sharing of the published article and lay summaries.

### Disclaimer

This manuscript has not been published previously and is not under consideration for publication elsewhere, in whole or in part. No part of this work has been copied from other sources.

### Use of artificial intelligence

AI-assisted large language model tools were used solely for language editing and grammatical refinement only, accessed during manuscript preparation (2026). All authors reviewed and approved the final manuscript and take full responsibility for its accuracy and integrity.
